# 

**Published:** 1842-05

**Authors:** 


### WESTERN JOURNAL

OF

### MEDICINE AND SURGERY.

### EDITORS.

DANIEL DRAKE, M. D.,

PROFESSOR OF PATHOLOGICAL ANATOMY AND CLINICAL MEDICINE IN THE MEDICAL INSTITUTE OF LOUISVILLE:

LUNSFORD P. YANDELL, M. D.,

PROFESSOR OF CHEMISTRY AND PHARMACY IN THE SAME:

THOMAS W. COLESCOTT, M. D.

To Subscribers.—Our receipts are now so trifling that we can no longer bear it. Subscribers must remit, and that without further delay. In the next number we shall commence the publication of the names of all that have not paid for the first year; and we shall keep the list standing as a remembrancer, omitting from time to time those that may pay up. Kentucky subscribers will remit Kentucky paper; Indiana subscribers may remit Indiana paper; Missouri and Illinois, Shawneetown; Mississippi, Arkansas, Louisiana, and Alabama, New Orleans paper. Subscribers in Tennessee may for the present remit Tennessee paper, if they can get no better.

In all cases where paper is remitted worse than that of the State in which the subscriber resides, and in all cases where subscribers, out of the States enumerated above, send us paper more than ten per cent discount, the excess shall be deducted from the remittance. But the good Ohio banks, Louisiana, Virginia, North and South Carolina, Indiana, and the good banks of the Eastern States, generally, will be received at par.

---

☞We have on hand a few copies of the Medical Journal, published by us in 1838, which we propose to send as premiums to all new subscribers to the present Medical Journal and all the present subscribers who are in arrears and may pay up *and one year in advance.*

It is a pamphlet of 275 pages and is filled with a great quantity and variety of interesting matter. It contains Judge Bibb's address on the laying of the corner stone of the Louisville Medicall Institute—an Introductory Lecture on its opening, by professor Caldwell—very interesting articles by Drs. Brent, Hardin, Bell, Rogers, Caldwell, &c. Those wishing to avail themselves of this offer should be in haste, as the supply of that number is limited, and the first orders will meet with the first attention.

---

### RECEIPTS FOR THE MEDICAL JOURNAL,

### FOR APRIL, 1842.

| | |
|---|---|
| Dr. W. A. F. Berry, Savannah, Tenn., | $5 00 |
| V. T, West, Princeton, Ia., | 5 00 |
| R. M. McFarland, Hebardsville, Ky., | 5 00 |
| J. Higgason, Somerville, Tenn., | 1 66 |
| L Hadden, Livingston, Ala, | 10 00 |
| R F. Brown, Sring Hill, Tenn., | 5 00 |
| J. F. Sowell, Athens, Ala,, | 5 00 |
| A. A. Burleson, Decatur, Ala., | 5 00 |
| W, H. Drain, Clarksville, Tenn., | 10 00 |
| M. Garst, Dayton, O., | 2 00 |
| W. McGuire, Waynesville, O, | 5 00 |
| B. G. Thornton, Batavia, N. Y., | 5 00 |
| J. B. Jones, Circleville, O.. | 7 00 |
| E L Mann, Lithopolis, O., | 5 00 |
| Strader, Cincinnati, O., | 5 00 |
| J G. Rogers, New Richmond, O, | 7 00 |
| J. R. Hunt, Springdale, O., | 8 50 |
| J B McFarland, Hamilton, O., | 5 00 |
| Drs. Walker & Harris, Richmond, Ky., | 10 00 |
| Dr. M. Pendleton, Stanford, Ky., | 5 00 |
| H. P. Sanderson, Crab Orchard, Ky | 5 00 |

